# Circular RNAs: Biogenesis, Function and Role in Human Diseases

**DOI:** 10.3389/fmolb.2017.00038

**Published:** 2017-06-06

**Authors:** John Greene, Anne-Marie Baird, Lauren Brady, Marvin Lim, Steven G. Gray, Raymond McDermott, Stephen P. Finn

**Affiliations:** ^1^Department of Histopathology and Morbid Anatomy, School of Medicine, Trinity College DublinDublin, Ireland; ^2^Department of Medical Oncology, Tallaght HospitalDublin, Ireland; ^3^Thoracic Oncology Research Group, Trinity Translational Medical Institute, St. James's HospitalDublin, Ireland; ^4^Department of Clinical Medicine, Trinity College DublinDublin, Ireland; ^5^Cancer and Ageing Research Program, Institute of Health and Biomedical Innovation, Queensland University of TechnologyBrisbane, QLD, Australia; ^6^Department of Medical Oncology, St. Vincent's University HospitalDublin, Ireland; ^7^HOPE Directorate, St. James's HospitalDublin, Ireland; ^8^Labmed Directorate, St. James's HospitalDublin, Ireland; ^9^Department of Histopathology, St. James's HospitalDublin, Ireland

**Keywords:** circRNAs, non-coding RNA, miRNA, diseases, cancer

## Abstract

Circular RNAs (circRNAs) are currently classed as non-coding RNA (ncRNA) that, unlike linear RNAs, form covalently closed continuous loops and act as gene regulators in mammals. They were originally thought to represent errors in splicing and considered to be of low abundance, however, there is now an increased appreciation of their important function in gene regulation. circRNAs are differentially generated by backsplicing of exons or from lariat introns. Unlike linear RNA, the 3′ and 5′ ends normally present in an RNA molecule have been joined together by covalent bonds leading to circularization. Interestingly, they have been found to be abundant, evolutionally conserved and relatively stable in the cytoplasm. These features confer numerous potential functions to circRNAs, such as acting as miRNA sponges, or binding to RNA-associated proteins to form RNA-protein complexes that regulate gene transcription. It has been proposed that circRNA regulate gene expression at the transcriptional or post-transcriptional level by interacting with miRNAs and that circRNAs may have a role in regulating miRNA function in cancer initiation and progression. circRNAs appear to be more often downregulated in tumor tissue compared to normal tissue and this may be due to (i) errors in the back-splice machinery in malignant tissues, (ii) degradation of circRNAs by deregulated miRNAs in tumor tissue, or (iii) increasing cell proliferation leading to a reduction of circRNAs. circRNAs have been identified in exosomes and more recently, chromosomal translocations in cancer have been shown to generate aberrant fusion-circRNAs associated with resistance to drug treatments. In addition, though originally thought to be non-coding, there is now increasing evidence to suggest that select circRNAs can be translated into functional proteins. Although much remains to be elucidated about circRNA biology and mechanisms of gene regulation, these ncRNAs are quickly emerging as potential disease biomarkers and therapeutic targets in cancer.

## Introduction

Non-coding RNAs (ncRNA), which include short microRNAs (miRNA), long non-coding RNAs (lncRNA) and circular RNAs (circRNA) make up 95% of total RNA in eukaryotic transcription, and are being increasingly appreciated to have an important function in gene regulation (Warner, 1999; Esteller, 2011; Jeck et al., 2013). circRNAs are generated from the backsplicing of exons, introns, or both to form exonic or intronic circRNAs (Jeck et al., 2013). The first examples of circRNA transcripts were first identified over 30 years ago, however at that time they were thought to represent errors in RNA splicing, but are now appreciated to have important functions in gene regulation (Sanger et al., 1976; Kos et al., 1986; Nigro et al., 1991; Cocquerelle et al., 1993; Jeck et al., 2013). It wasn't until recently that interest in circRNA research was re-established by Salzman et al. who identified circRNAs in RNA Sequencing (RNA-Seq) samples of cancer and non-cancer cell lines, and from patients with Acute Lymphoblastic Leukemia (ALL) (Salzman et al., 2012). This development has led to the identification of thousands of individual circRNAs that are endogenous to mammalian cells and are both highly stable and abundant *in vivo* compared to their linear counterparts (Jeck and Sharpless, 2014). More recently, advances in high-throughput sequencing, novel bioinformatics approaches and corresponding experimental validation, have proven that circRNAs actually represent a distinct class of ncRNAs (Jeck and Sharpless, 2014). Unlike linear RNA, the 3′ and 5′ ends in circRNA normally present in an RNA molecule have been joined together, forming a covalently closed continuous loop, which prevents degradation by RNA exonucleases. This confers stability on circRNAs and makes them very abundant in the cytoplasm (Jeck et al., 2013). It has been proposed that cellular levels of circRNAs may be regulated by either endonucleic activity or removal by exosomes (Lasda and Parker, 2016). Their high abundance, stability and evolutionary conservation between species suggests that they may have an important regulatory role and indeed, recent evidence suggests circRNAs appear to act as miRNA sponges, in part due to the competitive endogenous RNA (ceRNA) network (Hansen et al., 2013a). circRNAs have been shown to be tissue specific and to be expressed in pathological conditions, which has stimulated significant interest into their role in human disease and cancer (Salzman et al., 2013). Although their exact roles and mechanisms of gene regulation remain to be clarified, circRNAs may have potential as disease biomarkers and novel therapeutic targets.

## Biogenesis of circRNAs

Canonical eukaryotic pre-mRNA splicing is catalyzed by the spliceosomal machinery to remove introns and join exons leading to the formation of a linear RNA transcript with 5′–3′ polarity (Chen and Yang, 2015). Most circRNAs are produced during backsplicing, which does not follow the canonical 5′–3′ order, and is generally catalyzed by either the spliceosomal machinery or by group I and II ribozymes (Vicens and Westhof, 2014). circRNAs are distinct from their linear counterparts because they lack the usual terminal structures (e.g., 5′ cap or a polyadenylated (poly(A)) tail) due to their closed covalent bonds (Memczak et al., 2013). Inhibition of the canonical spliceosome using isoginkgetin, a pre-mRNA splicing inhibitor, reduces circRNA levels as well as the levels of the spliced linear transcript, providing evidence for a role for the spliceosome in circRNA biogenesis (Starke et al., 2015). The expression of circRNA does not always correlate with the expression level of the linear transcript from which the circRNA is derived, indicating that expression of circRNA is regulated and that the spliceosome must be able to discriminate between forward splicing, i.e., canonical linear splicing and backsplicing (Chen and Yang, 2015).

**Figure 1:**
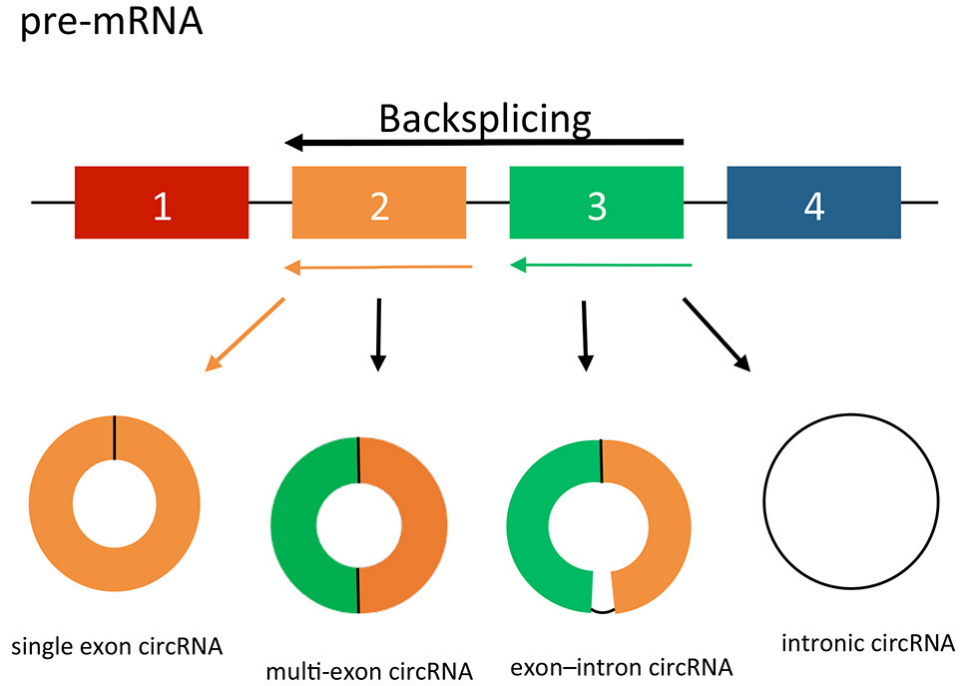
circRNA splicing. circRNAs are created by non-canonical splicing process known as “backsplicing.” A downstream splice donor is joined to an upstream splice acceptor. circRNAs can be exonic, intronic or a combination of both. Colored bars = exons. Black lines = introns.

circRNAs may arise from exons or introns leading to the formation of three different types of circRNAs; exonic, intronic and exon-intron circRNAs (Jeck et al., 2013). The formation of exonic circRNAs are a result of pre-mRNA splicing when the 3′ splice donor attaches to the 5′ splice acceptor forming an exonic circRNA (Figure 1; Jeck et al., 2013; Jeck and Sharpless, 2014). In some cases, this happens with a single exon, whereas in others the start of an upstream exon splices to the end of a downstream exon, with the intervening RNA circularized, producing circRNAs from multiple exons. Alternatively, if the intron between the exons is retained, the resulting circular transcript is referred to as exon–intron circRNA (Li Z. et al., 2015). Finally, intronic circRNAs can be produced from intron lariats that are resistant to degradation by de-branching enzymes (Jeck et al., 2013; Li Z. et al., 2015). Intronic circRNAs contain a single unique 2′–5′ linkage that distinguishes them from exonic circRNAs (Jeck and Sharpless, 2014). The formation of intronic circRNAs relies on GU-rich sequences near the 5′ splice site and C-rich sequences near the branch point (Zhang et al., 2013). During the backsplicing process, the two segments bind into a circle first, the exonic and intronic sequences in the binding part are cut out by the spliceosome with the remaining introns brought together to form intronic circRNA.

Recently, researchers have demonstrated that RNA binding-proteins (RBPs) may serve as regulatory activators or inhibitors in the formation of circRNAs in some conditions (Conn et al., 2015). This has been shown for the RBP that regulates muscle-blind protein (MBL) levels in the fly brain (Ashwal-Fluss et al., 2014). When levels are too high, RBPs bind to pre-mRNA and cause it to splice into circRNA, thereby preventing linear splicing and translation into MBL protein (Ashwal-Fluss et al., 2014; Meng et al., 2016). Further evidence for RBPs regulating circRNA levels comes from the Quaking (QKI) protein, which belongs to the STAR (signal transduction and activation of RNA) family of RBPs, and the ADAR (adenosine deaminases acting on RNA) protein (Conn et al., 2015). QKI effects pre-mRNA, mRNA turnover and translation, and has been implicated in a number of diseases including cancer (Chenard and Richard, 2008). QKI mediates regulation of circRNA biogenesis by binding to each intron flanking a circRNA and facilitates dimerization to form a looped structure which promotes circularization. ADAR is a regulatory RBP that is involved in RNA editing, and is essential for mammalian development. It has been implicated in decreasing circRNA production by weakening and editing RNA duplexes which decreases the likelihood of circularization (Rybak-Wolf et al., 2015).

Although, circRNAs are classified as ncRNA, it has recently been reported that circRNAs may be translated into protein, if there is an internal ribosome entry site (IRES) present (Granados-Riveron and Aquino-Jarquin, 2016; Schneider et al., 2016; Pamudurti et al., 2017). An IRES is an alternative means of translation initiation in eukaryotes that is independent of the 5′ cap structure and 3′ poly(A) tail recognition (Hernandez et al., 2004). Interestingly, the presence of an IRES might allow the formation of peptides or proteins from RNA in circular conformation by a rolling circle amplification (RCA) mechanism, where the circRNA contains no stop codon and the number of nucleotides composing the RNA is a multiple of three (Abe et al., 2015). Further evidence for translation of exonic circRNAs is from Wang et al., who developed a single exon minigene containing split GFP that could be efficiently backspliced to generate circRNA and directed the translation of an entire functional GFP protein (Wang and Wang, 2015). Similarly, Chen et al. have recently discovered 45 peptides that map to 46 circRNAs from 37 genes that have their corresponding proteins expressed in the human brain (Chen et al., 2016). Additionally, Legnini et al. have identified circ-ZNF609, which is translated into a protein in a splicing-dependent and cap-independent manner (Legnini et al., 2017). The resultant protein has a functional role in muscle differentiation in Duchenne muscular dystrophy by regulating myoblast proliferation.

## Detecting circRNAs

### RNA- sequencing

Advances in sequencing technology have enhanced the methods of detection of cirRNAs including deeper sequencing with longer read lengths, the development of better algorithms for mapping RNA and the use of enriched RNA libraries. Salzman et al., originally looking for genomic rearrangements in RNA-seq samples from patients with ALL, found there was an unexpected abundance of fragments in which two read pairs mapped to the same gene but were in the opposite order (Salzman et al., 2012). This method was further developed by scanning for out-of-order paired end reads for specific genes which then allowed for quantitative PCR (qPCR) validation in cancer and non-cancer cell lines (Salzman et al., 2012). The detection of circRNAs was further developed by Jeck et al. and Memczak et al. using bioinformatic analyses on RNA-seq libraries to identify specific back-splice junctions *de novo* (Jeck et al., 2013; Memczak et al., 2013).

circRNAs, uniquely, are linked by covalently joined bonds at their 5′ and 3′ ends, and having no polyA tail, are therefore able to avoid detection by standard molecular techniques including qPCR. One way to differentiate circRNAs from linear RNAs is by using a RNA exonuclease enrichment strategy. Ribonuclease R (RNase R) is a magnesium-dependent 3′ → 5′ exoribonuclease that digests essentially all linear RNAs but does not digest circRNA structures due to lack of free ends (Suzuki et al., 2006). Using this strategy, thousands of circRNAs have been identified that can contain one or more coding exons from linear messenger RNAs (mRNA) and can be hundreds to thousands of nucleotides in length (Guo et al., 2014). qPCR can be used to assess the relative abundance of circRNA across samples using divergent primers that are designed to specifically amplify and detect the backsplice junction (Jeck et al., 2013). Other methods which enrich for circRNAs in sequencing libraries, include the use of ribosomal RNA (rRNA) and polyA depletion techniques (Szabo and Salzman, 2016).

The majority of circRNAs have been identified from advances in high throughput sequencing technology. One of the first identified circRNA species called cANRIL (antisense non-coding RNA in the INK4 locus) was discovered when a genome-wide RNA assessment of circRNAs in cell lines was performed (Burd et al., 2010; Jeck et al., 2013). cANRIL is associated with the INK4a/ARF locus and is correlated with an increased risk of human atherosclerosis. Similarly, circRNAs have been detected by RNA-seq of human saliva samples, the first reported detection and validation of circRNAs in extracellular body fluids (Bahn et al., 2015).

However, there remains non-uniformity in RNA-Seq data sets as circRNA have relatively low abundance compared with their linear counterparts and some data sets were generated in the absence of RNase R enrichment (Szabo and Salzman, 2016). Other methods of detection include the use of high throughput microarrays using RNA samples enriched with RNase R (Wang et al., 2015). Zhong et al. used this method to identify circRNAs expressed in bladder cancer (Zhong et al., 2016). Using miRNA target prediction software, a circRNA-miRNA network was established by identifying specific miRNA binding sites for circRNAs called miRNA Response Elements (MRE), which allows the identification of potential miRNA sponge candidates (Bassett et al., 2014).

### Bioinformatic pipelines

A number of bioinformatic algorithms have been developed for identifying circRNAs. Salzman et al. developed an algorithm that detected circRNA based on the alignment of reads to a customized database of annotated exon boundaries (Salzman et al., 2012). This was further improved by adding a false discovery rate (FDR)-controlled filtration based on statistics of alignment quality scores (Salzman et al., 2013). Leading on from this, Memczak et al. developed their method using GT-AG splicing signals flanking exons as a *de novo* filter to identify circRNAs (Memczak et al., 2013). Further algorithms for mapping low-divergent sequences against a large reference genome have also been used to identify circRNAs using BWA-MEM and Segemehl software, which can identify circular junctions (Bassett et al., 2014; Hoffmann et al., 2014).

Circle Seq is an example of a bioinformatics pipeline that uses a biochemical approach for genome-wide identification of circRNAs and has led to the discovery of thousands of circRNA candidates (Danan et al., 2012). Total RNA from a human fibroblast cell line, Hs68, was first enriched for circRNAs by depletion of rRNA followed by RNase R treatment. High throughput sequencing was performed and the resulting reads were aligned to the human genome using a mapping algorithm, Mapsplice, which identifies back-splice junctions rather than identifying the exon order (Wang et al., 2010). Back-splice reads were identified using a segmented mapping approach and reads were significantly enriched in the RNase R treated samples compared to the control samples (no RNase R), thus allowing detection of circRNAs.

Gao et al. identified circRNAs from RNA-seq reads using a novel algorithm called circRNA Identifier (CIRI), which is based on paired chiastic clipping (PCC) signal detection using rRNA depleted and RNase R enriched libraries (Gao et al., 2015). CIRI detects junction reads with PCC signals after filtering is performed using paired-end mapping and GT-AG splicing signaling for the junctions that indicate a possible circRNA. This method has revealed the prevalence of non-exonic circRNAs containing intronic or intergenic circRNA fragments that are not expressed in known linear RNAs. Significantly, a large proportion of circRNA alternatively spliced exons cannot be detected in mRNAs and are enriched with binding sites of distinct splicing factors from those enriched in mRNA exons (Gao et al., 2016).

### Online databases

A number of online resources exist, which provides investigators access to online circRNA databases. CircBase provides online merged and unified data sets of circRNAs and the evidence supporting their expression can be accessed and scripts are available to identify known and novel circRNAs in sequencing data (Glazar et al., 2014). A similar database is Circ2Traits which provides a database of potential circRNAs associated with diseases in humans (Ghosal et al., 2013). CircInteractome (circRNA interactome) is a web-based tool, which maps for RBPs and miRNA binding sites on human circRNAs (Dudekula et al., 2016). It allows the researcher to do several functions including (i) identify potential circRNAs which can act as RBP sponges and (ii) design junction-spanning primers for specific detection of circRNAs of interest. circRNADb is another example of a circRNA database, which includes detailed information of the circRNA, including genomic information, exon splicing, genome sequence, IRES, and open reading frames (ORFs) (Chen et al., 2016).

Ongoing research into the role and function of circRNA has initially focused on their potential regulatory role. One such role is the ceRNA network and further bioinformatic pipelines have been developed to search for miRNA sponge candidates (Guo et al., 2014).

## CircRNAs as miRNA sponges

**Figure 2:**
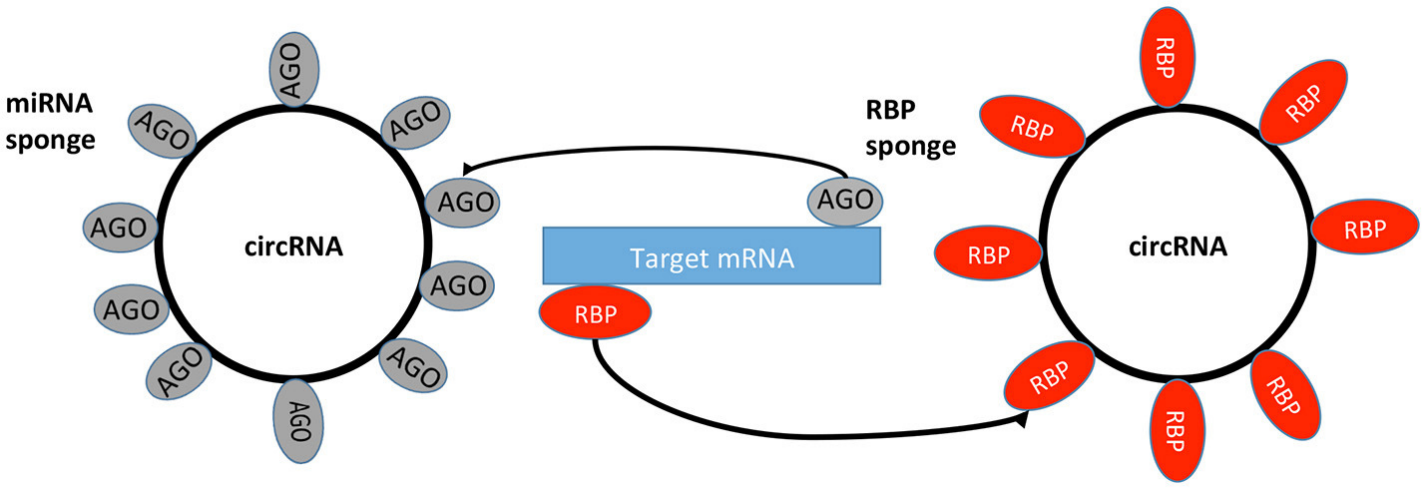
circRNA sponges. circRNAs can bind miRNA from its mRNA targets via RBPs and AGO proteins. RBP, RNA Binding Protein; AGO, Argonaute Protein; miRNA, microRNA; mRNA, messenger RNA.

miRNAs are a family of small ncRNAs that regulate a wide array of biological processes and may have an important role in regulating gene expression in cancer, where they act through the repression of downstream tumor-suppressive mRNA (Table 1; Romero-Cordoba et al., 2014). It has been theorized that circRNAs function as miRNA sponges through the ceRNA network (Taulli et al., 2013). The ceRNA hypothesis proposes that specific RNAs can impair miRNA activity through sequestration (Figure 2), thereby upregulating or downregulating miRNA target gene expression (Thomson and Dinger, 2016). An important function of miRNAs is the ability to regulate hundreds of targets, as well as to collectively function in networks in which a single target may have multiple MREs (Felekkis et al., 2010). Pipelines have been created using algorithms for miRNA target prediction, which allows for the identification of candidates for experimental validation (Glazar et al., 2014). However, some studies have shown that the general function of a circRNA is not as a miRNA sponge, and indeed only a small number of circRNAs have been identified as miRNA sponges to date (Salzman, 2016).

**Table 1:** Candidate circRNAs identified as miRNA sponges and their proposed functions.

**Putative miRNA sponges**	**Target miRNAs**	**Hypothesized miRNA function**
circRNA Sry (Hansen et al., 2013b)	miR-138	Tumor suppressor (Liu et al., 2016)
ciRS-7 (Hansen et al., 2013b)	miR-7	Tumor suppressor (Kalinowski et al., 2014) Oncogene (Hansen et al., 2013a)
circHIPK3 (Zheng et al., 2016)	miR-124	Tumor suppressor (Shi et al., 2013)
cirITCH (Li F. et al., 2015)	miR-7, miR-20a	Tumor suppressor (Kalinowski et al., 2014) Oncogene (Pesta et al., 2010)
circTCF25 (Zhong et al., 2016)	miR-103a-3p miR-107	Tumor suppressor (Zhong et al., 2016) Tumor suppressor (Zhong et al., 2016)
circPVT1 (Panda et al., 2016)	let-7	Cell senescence suppressor (Panda et al., 2016) Tumor suppressor (Boyerinas et al., 2010)

### ciRS-7

The most studied miRNA in circRNA research is miR-7, which regulates a variety of functions in disease and carcinogenesis such as cell development, proliferation and apoptosis (Li and Rana, 2014; Zhao et al., 2015). It has been detected in a number of malignancies, where it has been demonstrated to function as both an oncogene and a tumor suppressor including breast (Reddy et al., 2008), brain (Kefas et al., 2008), head and neck (Kalinowski et al., 2012), liver (Ning et al., 2014), melanoma (Giles et al., 2013), and lung cancer (Chou et al., 2010). Cerebellar degeneration-related protein 1 antisense RNA (CDR1as), also known as circular RNA sponge for miR-7 (ciRS-7), binds to miR-7 and regulates its function, providing the first evidence that circRNAs behave as miRNA sponges (Hansen et al., 2013a). ciRS-7 contains over 70 binding sites for miR-7 and is densely bound by Argonaute (AGO) proteins (Hansen et al., 2013b). AGO's are highly specialized small-RNA-binding modules and are considered to be the key components of RNA-silencing pathways (Ender and Meister, 2010). In diabetic mouse models, cirRS-7 overexpression has been shown to improve insulin secretion by inhibiting miR-7 function in pancreatic islet cells (Memczak et al., 2013; Xu et al., 2015). In hepatocellular cancer (HCC), ciRS-7 was over-expressed in cancer tissues with the corresponding miR-7 expression levels down-regulated when compared with adjacent non-tumor tissues (Yu et al., 2016).

### Sry circRNA

Sry circRNA, one of the original circRNAs to be detected, is encoded by the Sex Determining Region Y (*Sry*) gene that controls male sex determination in mammals. Sry circRNA exists primarily as a circular product that is predominantly localized to the cytoplasm (Koopman et al., 1990). Sry circRNA has been shown to act as a miR-138 sponge by Hansen et al. and has demonstrated *in vitro* to have 16 putative target sites for miRNAs (Capel et al., 1993; Hansen et al., 2013a).

### circHIPK3

Further evidence of a circRNA acting as a miRNA sponge comes from Zheng et al. who identified the circRNA candidate, circHIPK3, derived from exon2 of the *HIPK3* gene (Zheng et al., 2016). *In vitro* studies showed that silencing circHIPK3 in cancer cell lines caused a significant decrease in cancer cell growth (Zheng et al., 2016). Using luciferase screening, they observed that circHIPK3 could bind to multiple miRNAs, including the well-known tumor suppressor miR-124 (Wang Y. et al., 2016). It appears that circHIPK3 has a regulatory role as a modulator of cell growth by sponging multiple miRNAs in human cells.

### cirITCH

A recently discovered circRNA, called cirITCH, similarly acts as a miRNA sponge via miR-7 and miR-20a. It increases the level of *ITCH*, a protein coding gene, which provokes ubiquitin-mediated Dvl2 degradation and inhibits canonical Wnt signaling leading to an overall anti-tumor effect (Li F. et al., 2015).

## Exosomal circRNAs

Due to their high degree of stability and their resistance to exonuclease degradation, circRNAs may accumulate in cells if their levels are not adequately controlled by cellular mechanisms (Conn et al., 2015). One such mechanism is the excretion of circRNAs from cells via extracellular vesicles (EVs). EVs are membrane-bound vessels that are released from cells and can contain cellular components, including proteins, lipids, and RNA. Different EV types, including exosomes, have been characterized on the basis of their biogenesis or release pathways. Lasda et al. showed that circRNAs could be detected in EVs, including ciRS-7, suggesting that cells may use EVs to transport circRNAs to communicate to other cells, possibly as part of the ceRNA network (Lasda and Parker, 2016).

Exosomes are small membrane vesicles of endocytic origin secreted by most cell types that contain proteins, mRNA, miRNA and more recently have been shown to contain circRNAs by Li Y. et al. (2015). circRNA transcripts were identified by RNA-seq analyses of MHCC-LM3 liver cancer cells and cancer cell-derived exosomes. A higher percentage of circRNAs compared to linear RNAs were detected in exosomes compared to producer cells. circRNAs within exosomes have been shown to be stable in serum, and remain intact and circularized after RNAse R treatment. The presence of circRNAs within exosomes was also confirmed in a panel of cancer cell lines, including colon, lung, stomach, breast and cervical cancer (Li Y. et al., 2015). Further RNA-seq was performed on exosomes derived from the serum of patients with colorectal cancer (CRC). In these exosomes, the expression levels of circ-KLDHC10 was significantly increased in the serum of patients with CRC compared to normal healthy controls serum (Li Y. et al., 2015).

## circRNAs and human disease

Although much remains to be learned about circRNA biology and their mechanisms of gene regulation, they are quickly emerging as potential therapeutic targets in a very similar fashion to their other non-coding relatives, miRNAs (Valdmanis and Kay, 2013). circRNAs have been shown to be both abundant and stable and are now being studied extensively in human diseases (Table 2).

**Table 2:** circRNAs identified in human diseases.

**Disease**	**CircRNA**	**Function**
Ischaemic Heart Disease	cANRIL	Repression of the INK4A/ARF locus associated with an increased risk of atherosclerosis (Burd et al., 2010).
	cZNF292	Regulated by hypoxia in endothelial cells and controls angiogenesis (Boeckel et al., 2015).
	hsa_circ_0124644	Upregulated in coronary artery disease (Zhao et al., 2017).
Alzheimer's Disease	ciRS-7	Up-regulates UBE2A that aids the clearance of amyloid peptides (Lukiw, 2013).
	CircPVT1	Cell senescence suppressor (Panda et al., 2016).
Diabetes	ciRS-7	Inhibits miR-7 function in islet β cells, which in turn improves insulin secretion (Lukiw, 2013).

### Cardiovascular disease

Ischaemic heart disease (IHD) is a leading cause of morbidity and mortality in the developed world. One of the first identified circRNAs, cANRIL, is associated with common single-nucleotide polymorphisms (SNPs). These have been predicted to affect cANRIL splicing leading to repression of the *INK4A/ARF* locus, which is associated with an increased risk of atherosclerosis (Burd et al., 2010). Interestingly hypoxia, a known risk factor for atherosclerosis, is a key stimulus for angiogenesis and is associated with significant regulation of circRNAs (Boeckel et al., 2015). This is supported by evidence from Boeckel et al. who have shown that the circRNA, cZNF292, is regulated by hypoxia in endothelial cells and controls angiogenesis. circRNAs are highly expressed in human heart tissue and are associated with key cardiac genes including *Titin* (TTN), *RYR2*, and *DMD* (Tan et al., 2017). Further evidence for the role of circRNA in cardiovascular disease is supported by research from Jakobi et al. who performed RNA-seq on adult murine hearts and identified a further 575 candidate cardiac circRNAs (Jakobi et al., 2016). Some of these candidates coincide with disease-associated gene loci that have been previously linked to cardiovascular disease. Importantly, circRNAs have been identified in peripheral blood samples from patients with IHD and hsa_circ_0124644 has been found to be significantly upregulated in these patients compared to healthy controls (Zhao et al., 2017).

### Alzheimer's disease

Initial studies have shown that circRNAs are highly expressed in the brain (Zheng et al., 2016) and may participate in regulating synaptic function and neural plasticity (You et al., 2015). This is further supported by research from Lukiw et al., who detected a mis-regulated miR-7-circRNA system in the sporadic Alzheimer's disease (AD) hippocampal CA1 region of the brain (Lukiw, 2013). Up-regulation of miR-7 in the AD brain, due to a deficiency in ciRS-7, down-regulates several AD-relevant mRNA targets and their expression, including the ubiquitin conjugase (UBE2A) protein. UBE2A a central effector in the ubiquitination cycle, that aids the clearance of amyloid peptides via phagocytosis. It is depleted in sporadic AD brain and contributes to amyloidogenesis.

Further evidence for an association with aging comes from the detection of senescence-associated circRNAs (Panda et al., 2017). Cellular senescence is a state of indefinite growth arrest triggered by the exposure of cells to stress-causing stimuli and has been associated with disease processes such as sarcopenia, arthritis, diabetes, neurodegeneration and cancer (Campisi, 2013). circPVT1 has been shown to act as a senescence suppressor in proliferating fibroblasts and appears to sequester let-7, which has in role in neurodegeneration, seen in patients with AD (Lehmann et al., 2012; Panda et al., 2016).

### Diabetes

Diabetes is associated with significant long-term health consequences and earlier methods of detection and better treatments are required. Xu et al. have shown overexpression of miR-7 in transgenic mouse β cells results in diabetes. Similarly, it has also been shown that overexpression of ciRS-7 inhibits miR-7 function in islet β cells, which in turn improves insulin secretion (Xu et al., 2015). The potential gene targets for miR-7 were identified from bioinformatics analyses and include *Myrip* (regulates insulin granule secretion) and *Pax6* (enhances insulin transcription).

## circRNAs and cancer

Recent evidence suggests circRNAs may have a role in the development and progression of cancer (Table 3; Burd et al., 2010; Wang F. et al., 2016; He et al., 2017). The fact that circRNAs appear to behave as miRNA sponges, has only increased this interest (Kent and Mendell, 2006). The expression of miRNA is dysregulated in human cancer through various mechanisms, including amplification or deletion of miRNA genes, abnormal transcriptional control of miRNAs, dysregulated epigenetic changes and defects in the miRNA biogenesis machinery (Peng and Croce, 2016). Zheng et al. have identified over 27,000 circRNA candidates from the sequencing data of six human normal tissues (brain, lung, heart, stomach, colon, and liver) and seven human cancers (bladder cancer, breast cancer, colorectal cancer, hepatocellular carcinoma, gastric cancer, kidney clear cell carcinoma and prostate adenocarcinoma) (Zheng et al., 2016). circRNAs appear to be more often downregulated in tumor tissue compared to normal tissue and this may be due to (i) errors in the back-splice machinery in malignant tissues, (ii) degradation of circRNAs by deregulated miRNAs in tumor tissue, or (iii) increasing cell proliferation leading to a reduction in circRNAs (Scotti and Swanson, 2016).

**Table 3:** Cancer associated circRNAs.

**Cancer type**	**circRNA**	**Up/Down-regulation**	**Tissue Type**
Gastric Cancer (Li P. et al., 2015)	hsa_circ_002059	Down	Tumor tissue, Plasma
Colon Cancer (Bachmayr-Heyda et al., 2015; Huang et al., 2015; Dou et al., 2016)	circ6229 cirITCH	Down Down	Tumor tissue Tumor tissue
Bladder Cancer (Zhong et al., 2016)	circFAM169A circTRIM24 circTCF25 circZFR circPTK2 circBC048201	Down Down Up Up Up Up	Tumor tissue
Hepatocellular Cancer (Qin et al., 2016; Shang et al., 2016; Yu et al., 2016)	hsa_circ_0001649 ciRS-7 hsa_circ_0005075	Down Up Up	Tumor tissue Tumor tissue Tumor tissue
Oesophageal Cancer (Li F. et al., 2015)	cirITCH	Down	Tumor tissue
Lung Cancer (Wan et al., 2016)	cirITCH	Down	Tumor tissue

### Gastric cancer

Cancer of the stomach remains a common cause of cancer-related deaths in the world, despite a marked decline in the incidence in many developed nations. Li et al. have identified hsa_circ_002059, as a potential circRNA associated with gastric cancer (Li P. et al., 2015). Interestingly, this circRNA was found to be downregulated in gastric tumor tissue compared to normal controls. Furthermore, hsa_circ_002059 was detectable in plasma samples of patients with gastric cancer and found to be significantly downregulated compared to healthy controls. It was also associated with clinical and pathological features such as stage, distant metastases, gender and age (Li P. et al., 2015). Similarly, Chen et al. have identified circPVT, which is upregulated in gastric cancer tissues and promotes cell proliferation by sponging members of the miR-125 family (Chen et al., 2017).

### Colorectal cancer

CRC is the third most commonly diagnosed cancer and third leading cause of cancer death in both men and women. Bachmayr-Heyda et al. analyzed circRNA expression levels in clinically normal mucosa and tumor tissue samples from patients with CRC (Bachmayr-Heyda et al., 2015). circRNAs were found to be generally downregulated. Five circRNAs were randomly selected for validation, with four of the five circRNAs showing reduced expression in cancer compared to normal colon mucosa tissues (with only one significantly altered: circ6229). In a study of KRAS mutated CRC, which is associated with resistance to anti-EGFR (epidermal growth factor receptor) therapies, Duo et al. showed circRNA expression was more likely to be downregulated in KRAS-mutant colon cancer cell lines compared to wild-type (Dou et al., 2016). Similarly, cirITCH, has also been found to be downregulated in colorectal tissues compared to normal tissue (Huang et al., 2015). cirITCH acts as a miRNA sponge and increases the level of ITCH. The *ITCH* gene is involved in the Wnt/β-catenin pathway, which is associated with the onset and progression of CRC (Ye et al., 2015).

### Bladder cancer

Bladder cancer arises from the epithelial lining of the urinary bladder and is the ninth most common cause of cancer globally. circRNAs have also been identified in bladder cancer using high throughput microarrays (Zhong et al., 2016). Using this method, Zhong *el al*. identified two circRNAs that were significantly downregulated (circFAM169A, circTRIM24) and four that were significantly upregulated (circTCF25, circZFR, circPTK2, and circBC048201) in bladder carcinoma compared with adjacent non-tumor tissues. Furthermore, circTCF25 was shown to function as a miRNA sponge by down-regulating miR-103a-3p and miR-107 in cancerous tissue, leading to increased CDK6 expression. which is associated with the development of cancer (Zhong et al., 2016).

### Hepatocellular carcinoma (HCC)

Hepatocellular carcinoma (HCC) is a primary malignancy of the liver and occurs predominantly in patients with underlying chronic liver disease and cirrhosis. circRNAs have been shown to be dysregulated in HCC compared to normal tissues (Zheng et al., 2016). Qin et al. have identified hsa_circ_0001649 in HCC and found it to be significantly downregulated compared to paired adjacent normal liver tissues (Qin et al., 2016). In contrast to this, Shang et al. have identified a second circRNA, hsa_circ_0005075, to be significantly upregulated in HCC compared to normal tissues (Shang et al., 2016). Similarly, Yu et al. examined the previously discovered ciRS-7 in HCC, and found its expression upregulated in HCC tumor tissue compared with adjacent non-tumor tissues (Yu et al., 2016). As ciRs-7 is a known miR-7 sponge, further investigations, which included knockdown of ciRs-7 and overexpression of miR-7, found that ciRS-7 acts as an oncogene partly through targeting miR-7 in HCC.

### Other cancers

Other studies have identified reduced expression of circRNAs in oesophageal and lung cancer tissues compared to peritumoural and normal tissues (Li F. et al., 2015; Wan et al., 2016). Profiling for circRNA expression has also been performed in other upper gastrointestinal cancers including pancreatic cancer, showing significant dysregulation compared to normal tissue (Qu et al., 2015). Upregulation of circHIPK3, a proposed miRNA sponge, has been identified in cancerous tissues including prostate and renal cell carcinoma (RCC) (Zheng et al., 2016). Furthermore, targeting circRNAs associated with known cancer related genes may identify novel methods to overcome therapy related resistance (Li F. et al., 2015; Wan et al., 2016). Recently, investigators have shown that circHIAT1 functions as a metastatic inhibitor to suppress androgen receptor (AR)-enhanced RCC cell migration and invasion and that targeting AR could suppress RCC cell progression via increasing circHIAT1 expression (Wang et al., 2017).

### Mechanisms of circRNAs in cancer

As described above, a number of mechanisms have been postulated for circRNAs in cancer, with most investigators focused on their function as miRNA sponges through the ceRNA network (Taulli et al., 2013). It has been proposed that circRNA regulate gene expression at the transcriptional or post-transcriptional level by interacting with miRNAs (Hansen et al., 2013a; Wang F. et al., 2016). An important function of miRNAs is the ability to regulate hundreds of targets (Felekkis et al., 2010). One of the most studied miRNAs in the ceRNA network is miR-7. Emerging evidence indicates that cirs-7 regulates miR-7 expression. miR-7 can directly downregulate oncogenes, including epidermal growth factor receptor (EGFR), P21-activated kinase-1 (Pak1), insulin receptor substrate-1 (IRS-1), phosphoinositide 3-kinase catalytic subunit delta (PIK3CD), and mammalian target of rapamycin (mTOR) (Dong et al., 2017). It has also been demonstrated that RBPs may serve as regulatory activators or inhibitors in the formation of circRNAs in some conditions (Conn et al., 2015). RBPs effect pre-mRNA, mRNA turnover and translation, and have been implicated in a number of diseases including cancer (Chenard and Richard, 2008).

Interestingly, chromosomal translocations have been shown to generate aberrant fusion-circRNAs (f-circRNAs) in cancer (Guarnerio et al., 2016). Oncogenic fusion proteins are formed by the joining together of two otherwise-separated genes, leading to the formation of a fusion gene and the generation of a mutated protein which can result in the onset and progression of cancer (Meyerson et al., 2010; Chin et al., 2011; Greuber et al., 2013). It has been proposed that f-circRNAs are formed by unrelated intronic sequences coming together, which leads to new events of backsplicing (Barrett et al., 2015). Guarnerio et al. confirmed the presence of f-circRNAs in lung cancer for the *EML4/ALK1* translocation and the *EWSR1/FLI1* translocation associated with Ewing Sarcoma in samples using RNA-seq (Guarnerio et al., 2016). These f-circRNAs were validated in the appropriate cell line models and were shown to exert both proto-oncogenic and pro-proliferative effects promoting cancer development. This was in part due to triggering the activation of the PI3K and MAPK signal transduction pathways (Guarnerio et al., 2016). f-circRNAs also appear to confer resistance to therapy, with *in vitro* studies showing f-circM9 conferring protection to leukaemic cells upon treatment with arsenic trioxide (ATO) (Guarnerio et al., 2016). Indeed, it may prove more beneficial to target f-circRNAs instead of circRNAs to overcome therapy resistance.

Furthermore, examining pathways involved with carcinogenesis could allow investigators identify circRNAs that could be used as biomarkers. For instance, circRNAs appear to have a role in regulating the Wnt/β-catenin pathway (Huang et al., 2015). Wnt signaling has been shown to contribute to human tumor progression (Padala et al., 2017). If circRNAs are confirmed to regulate this pathway, circRNAs could also be targeted in order to interrupt this pathway and inhibit tumor growth and development. Other researchers have shown that overexpression of ciRS-7 in cancer tissues permitted inhibition of miR-7 and subsequent activation of EGFR and RAF1 oncogenes, again, strengthening the argument that circRNAs play a role in cancer initiation and development (Weng et al., 2017). PI3K/ATK and Raf/MEK/ERK pathways may be of interest in the future for investigating potential regulatory circRNAs.

## Future directions

Ongoing research is furthering our understanding of the complex circRNA network in non-cancer and cancer conditions, with early data suggesting roles in cancer initiation, progression and therapy resistance. The fact that certain circRNAs appear to be specific to certain diseases, are stable and have a regulatory function has led to further research into the use of circRNAs as potential diagnostic, prognostic biomarkers and therapeutic targets. Reliable sensitive and specific biomarkers are needed to aid researchers and clinicians in the early diagnosis of disease, identification of high risk populations and to develop targeted therapies and assess response. Blood-based analyses, often described as the “liquid biopsy” are of interest due to the potential for regular minimally invasive screening of patients (Abbosh et al., 2017). These blood-based analyses may also allow researchers to monitor responses to treatment, to detect resistance to treatment or to detect early recurrence in real time. miRNAs have become useful disease markers and current research is investigating replacement therapy using miRNA mimics, which has moved into promising early clinical trials (Reid et al., 2013). In the future it may be possible to use circRNA mimics in the same way to treat both non-cancer and cancer conditions. Though research in circRNAs is still relatively early, this family may provide the next generation of precision personalized medicine, given their tissue specificity, stability and suitability in “liquid biopsies.”

## Conclusion

To date, thousands of circRNAs have been detected and associated with a number of specific functions. Improving our understanding of their molecular mechanisms of action and further characterization of the complex circRNA network will provide important novel insights into cellular development human disease and carcinogenesis.

## Author contributions

All authors listed, have made substantial, direct and intellectual contribution to the work, and approved it for publication.

### Conflict of interest statement

The authors declare that the research was conducted in the absence of any commercial or financial relationships that could be construed as a potential conflict of interest.
